# Inter-Fraction Tumor Volume Response during Lung Stereotactic Body Radiation Therapy Correlated to Patient Variables

**DOI:** 10.1371/journal.pone.0153245

**Published:** 2016-04-06

**Authors:** Samer Salamekh, Yi Rong, Ahmet S. Ayan, Xiaokui Mo, Terence M. Williams, Nina A. Mayr, John C. Grecula, Arnab Chakravarti, Meng Xu-Welliver

**Affiliations:** 1 Department of Radiation Oncology, The Ohio State University Wexner Medical Center, Columbus, Ohio, United States of America; 2 Department of Radiation Oncology, University of California Davis, Sacramento, California, United States of America; 3 Center for Biostatistics, The Ohio State University Wexner Medical Center, Columbus, Ohio, United States of America; 4 Department of Radiation Oncology, University of Washington, Seattle, Washington, United States of America; North Shore Long Island Jewish Health System, UNITED STATES

## Abstract

**Purpose:**

Analyze inter-fraction volumetric changes of lung tumors treated with stereotactic body radiation therapy (SBRT) and determine if the volume changes during treatment can be predicted and thus considered in treatment planning.

**Methods and Materials:**

Kilo-voltage cone-beam CT (kV-CBCT) images obtained immediately prior to each fraction were used to monitor inter-fraction volumetric changes of 15 consecutive patients (18 lung nodules) treated with lung SBRT at our institution (45–54 Gy in 3–5 fractions) in the year of 2011–2012. Spearman's (ρ) correlation and Spearman's partial correlation analysis was performed with respect to patient/tumor and treatment characteristics. Multiple hypothesis correction was performed using False Discovery Rate (FDR) and q-values were reported.

**Results:**

All tumors studied experienced volume change during treatment. Tumor increased in volume by an average of 15% and regressed by an average of 11%. The overall volume increase during treatment is contained within the planning target volume (PTV) for all tumors. Larger tumors increased in volume more than smaller tumors during treatment (q = 0.0029). The volume increase on CBCT was correlated to the treatment planning gross target volume (GTV) as well as internal target volumes (ITV) (q = 0.0085 and q = 0.0039 respectively) and could be predicted for tumors with a GTV less than 22 mL. The volume increase was correlated to the integral dose (ID) in the ITV at every fraction (q = 0.0049). The peak inter-fraction volume occurred at an earlier fraction in younger patients (q = 0.0122).

**Conclusions:**

We introduced a new analysis method to follow inter-fraction tumor volume changes and determined that the observed changes during lung SBRT treatment are correlated to the initial tumor volume, integral dose (ID), and patient age. Furthermore, the volume increase during treatment of tumors less than 22mL can be predicted during treatment planning. The volume increase remained significantly less than the overall PTV expansion, and radiation re-planning was therefore not required for the purpose of tumor control. The presence of the studied correlations suggests that the observed volumetric changes may reflect some underlying biologic process rather than random fluctuations.

## Introduction

Stereotactic body radiation therapy (SBRT) or stereotactic ablative body radiotherapy (SABR) has become a highly effective radiation regimen for both early stage primary non-small cell lung cancer (NSCLC) and oligometastatic cancers to the lung. In this procedure, highly conformal and precise radiation doses of 50–60 Gy are delivered in 3–5 fractions to a malignant lung nodule, resulting in outstanding local control rate (>90% for early stage lung nodules).[1–4]

Due to the very high dose delivered in each SBRT fraction, the procedure requires robust patient immobilization, reproducible setup, accurate target delineation and sharp dose fall off outside the treatment target to avoid increased toxicity associated with elevated dose deposition in normal tissues. On-board kilo-voltage CBCT (kV-CBCT) can be used to improve the reproducibility of treatment setup as well as monitor tumor volume changes during treatment and evaluate the need for adaptive radiotherapy (ART) re-planning.[5–9]

Conventionally fractionated radiotherapy of lung nodules generally results in a time-dependent decrease in tumor volume during treatment.[10, 11] It was initially assumed that negligible inter-fraction tumor volume changes would be observed during the short SBRT treatment time; however, several recent studies have noted both decreases as well as increases in inter-fraction volumes.[12–18]

There however remains significant ambiguity whether these changes are significant and/or biologically meaningful. One source of uncertainty is that repeat imaging and volumetric analysis may result in small fluctuations in volume due to physiologic changes or imaging methodology, which may occur even in the absence of any treatment. Assuming volumetric calculations are accurate and not due to imaging artifact, it is unclear whether the changes occur in a random fashion or truly reflect the tumor’s response to radiation. This distinction is important because, if the inter-fraction changes reflect a biologic tumor response, they can be used as predictive marker, studied to better understand SBRT radiobiology, and applied to adaptive treatment planning.

We hypothesize that if strong correlations can be found between the observed tumor volume changes and the radiation delivered or other patient parameters known at the onset of treatment, it would provide support that the inter-fraction volumetric changes represent a biologic response rather than random volume fluctuations. Furthermore, the correlations may be used to understand the cause of these changes and better establish a prediction model for future patients. To the best of our knowledge, there are no studies showing that inter-fraction volumetric changes during lung SBRT treatment are related to other variables known at onset of treatment.

## Materials and Methods

### Patients and treatment delivery protocol

An IRB-approved retrospective study was performed on 15 consecutive patients receiving lung SBRT treatment with kV-CBCT imaging between November 1, 2011 and June 1, 2012. A total of 20 lung nodules were treated in these patients; however, only 18 nodules could be unambiguously delineated on kV-CBCT (Table 1).

**Table 1:** Patient parameters examined in the study.

Patient	Neoplasm	Age	Tumor Location	Volume of Treated Lung (mL)	ITV Volume (mL)	GTV Volume (mL)	Histology	Prescribed Dose per Fraction (Gy)	Number of Fractions
1	I	85	Right Lung	1447	5	1.9	Unknown	10.0	5
2	II	80	Left Lung	863	72.8	47.5	Squamous cell	18.0	3
3	III	80	Right Lung	1375	19.2	11.5	Adenocarcinoma	10.0	5
4	IV	57	Right Lung	1878	22.8	16.9	Squamous	17.0	3
5	V	77	Left Lung	1938	82.9	46.6	Adenocarcinoma	10.0	5
6	VI	72	Left Lung	1333	10	6.3	Squamous cell	10.0	5
	VII		Right Lung	1814	2.6	0.7	Squamous cell	10.0	5
7	VIII	66	Right Lung	2057	5.5	3.2	Adenocarcinoma	9.0	5
8	IX	86	Left Lung	1071	77.2	57.1	Adenocarcinoma	10.0	5
9	X	82	Left Lung	1695	7.3	5.3	Squamous cell	10.0	5
	XI		Left Lung	1695	0.9	0.5	Unknown	10.0	5
	XII		Right Lung	1658	3.1	1.8	Unknown	10.0	5
10	XIII	63	Right Lung	2172	31	21.7	Squamous cell	10.0	5
11	XIV	70	Right Lung	1125	27	21.2	Adenocarcinoma	9.5	5
12	XV	64	Left Lung	1676	10.2	5.0	Atypical cells	10.0	5
13	XVI	66	Right Lung	1408	16.7	9.9	Adenocarcinoma	18.0	3
14	XVII	70	Right Lung	822	18.5	17.6	Adenocarcinoma	9.0	5
			^1^Left Lung	602	16.5	10.6	Adenocarcinoma	9.0	5
15	XVIII	62	Right Lung	1612	4.4	3.6	Adenocarcinoma	18	3
			^1^Left Lung	1127	3.5	2.1	Adenocarcinoma	10	5

^1^Not included in the study because of significant fibrosis/imaging artifacts.

During simulation, each patient had one free-breathing CT and one 4D-CT using the Siemens SOMATOM CT scanner (Siemens Healthcare, Erlangen, Germany). Varian Respiratory Position Management (RPM) system (Varian Medical Systems, Palo Alto, USA) was used to trace breathing motion and generate 4D-CT scans. 4D-CT was co-registered with the free-breathing CT and internal target volumes (ITV) where generated by combining the gross tumor volume (GTV) generated during each phase of the breathing cycle. Target delineation was performed by the attending physician using Eclipse treatment planning system (Varian Medical Systems, Palo Alto, CA, USA) with a standard CT lung window. A 5–8 mm margin was used to expand ITV to planning target volume (PTV), with greater margins often given in the superior-inferior direction per RTOG guidelines.

Treatment plans were generated using various planning techniques, including 7–10 field non co-planer conformal technique and intensity modulated radiotherapy (IMRT). TrueBEAM™ system (Varian Medical Systems, Palo Alto, CA) was used to acquire pre-treatment CBCTs for tumor position verification and treatment plan delivery. The CBCT images were acquired using a preset thorax technique (125 kV voltage and 550 mAs). The images have an in-plane resolution of 512 x 512 pixels, 2 mm slice thickness, and 17 cm longitudinal coverage. CBCT images of each patient were co-registered with the planning CT.

### Ethics Statement

This study was approved by the Cancer Institutional Review Board in the Office of Responsible Research Practices. In addition, data pertaining to this study were de-identified prior to analysis.

## Contouring Protocol

**Fig 1:**
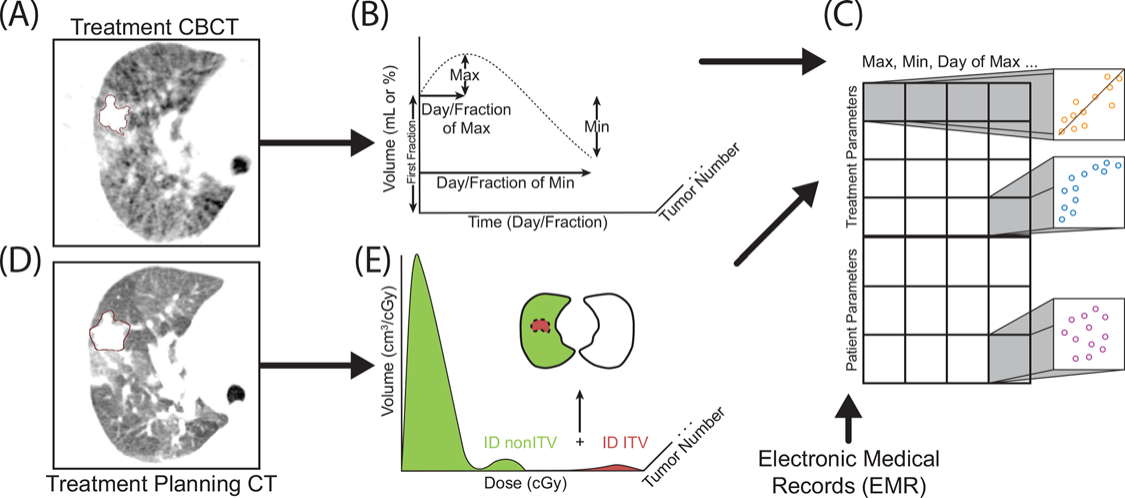
Summary of the analysis method. CBCTs were contoured by a single researcher and then verified by an attending physician (A), and the time course of each tumor was simplified to 7 parameters (B). Treatment planning CT contours (D) were used to determine the integral dose (ID) of structures listed in Table 2 (E). Each square in C represents a Spearman correlation analysis pair between inter-fraction volumes (B), EMR data, and treatment parameters (E).

To minimize inter-observer variability, tumors on all CBCTs were contoured using default lung window settings by a single researcher, and then verified by an attending physician (Fig 1A). The target volumes including GTV, ITV, PTV were contoured and confirmed by the treating physician on the free breathing CT and 4DCT during simulation (Fig 1D).

**Fig 2:**
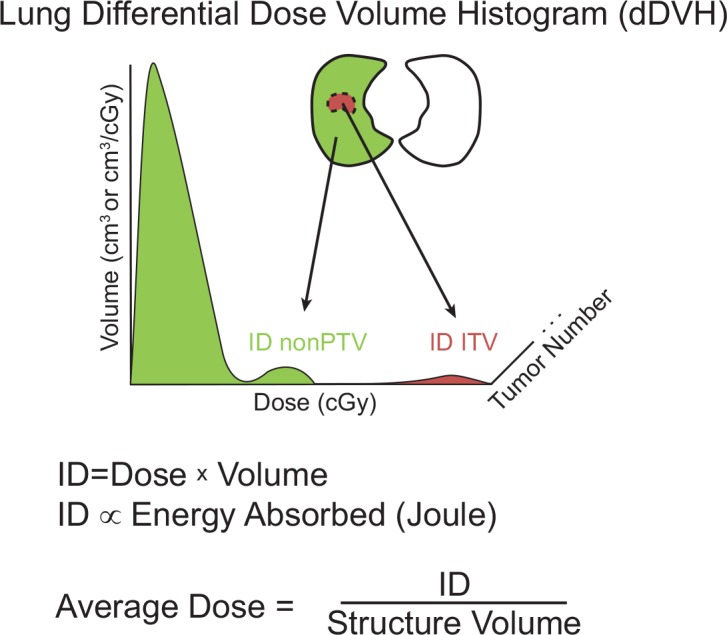
Integral dose calculations. Differential dose volume histogram (dDVH) of the ITV and non-PTV contours was extracted from Eclipse treatment planning software with dose increments of 0.1cGy. Tumor (ITV) volume in brown and healthy lung tissue (non-PTV) shown in green. The integral dose (ID) was calculated by summing the product of the dose and volume at each dose. Average dose was calculated by dividing the ID by structure volume.

The planning CT tumor volumes and the CBCT tumor volumes, as well as their corresponding differential dose-volume histograms (dDVH) in 0.1 cGy increments were exported into Matlab (MathWorks, Natick, MA) for further analysis. The integral dose (ID), in units of cGy*cm^3^, was then calculated (Fig 1E and Fig 2). The average dose, in units of cGy, was determined by dividing the ID by the structure volume and cross-compared with the average dose calculated by the treatment planning software.

### Statistical analysis

The inter-fraction volume changes of each tumor were simplified to 7 parameters: first CBCT volume (FCV), maximum volume (within the treatment course), minimum volume (within the treatment course), days to maximum volume, fraction to maximum volume, days to minimum volume, and fraction to minimum volume (Table 2). Correlation analysis between each volume parameter (Fig 1B) and treatment planning data (Fig 1E) as well as tumor/patient characteristics was then performed (Fig 1C). The correlation between numerical parameters was determined by Spearman’s ρ that is sensitive to both linear and non-linear correlations. Partial Spearman’s correlation analysis between two variables while controlling for a third variable was also performed. The p-values were adjusted by linear step-up method of Benjamini and Hochberg to control false discovery rate (FDR).[19] Q-values < 0.05 are considered significant, reported p-values represent statistical analysis without multiple hypothesis correction.

**Table 2:** Parameters examined in the study.

CBCT Volume Parameters	Patient/Tumor Parameters	Treatment Parameters
Minimum Volume	Age	Prescribed Dose
Maximum Volume	Lung Volume	ITV Average Cumulative Dose
Days to Minimum Volume	Histology	non-PTV Average Cumulative Dose
Fractions to Minimum Volume	GTV Volume	ITV Average Dose per Fraction
Days to Maximum Volume	ITV Volume	non-PTV Average Dose per Fraction
Fraction to Maximum Volume		ITV ID per Fraction
First CBCT Volume (FCV)		non-PTV ID per Fraction

CBCT, cone-beam CT; PTV, planning target volume; ITV, internal target volume; ID, integral dose

The volumes were compared both as absolute volume change in milliliters, denoted as “(mL)”, and as values normalized to the FCV, denoted as “(%)”. Linear regression fitting was performed on some of the data where noted and an r value as well as slope is reported. The effect of tumor histology was studied with a two-tailed student's t-test. Data analysis was performed by using SAS 9.4 (SAS, Inc; Cary, NC) and Matlab (MathWorks, Natick, MA).

## Results

### Patient characteristics and CBCT tumor contours

The patients included in the study had an average age of 73 (range: 57 to 86 years) and were treated with a total of 45–54 Gy in 3 or 5 fractions (Table 1). Patients were treated every other day with the exception of weekends (S1 Fig). The average FCV is 13.9 mL (range: 0.41 to 58.2 mL) (S1 Fig) and the average ipsilateral lung volume for these patients is 1535 mL (range: 822 to 2172 mL).

**Fig 3:**
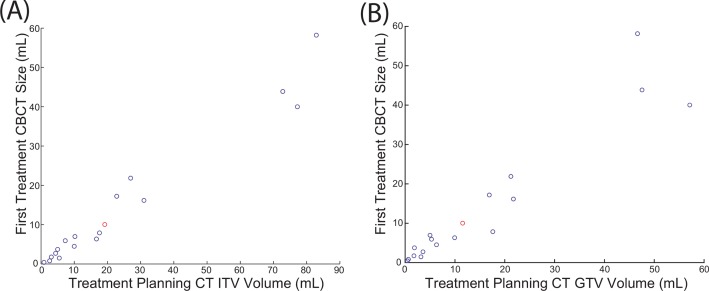
Contour volume of the treatment planning CT relative to first CBCT volume. FCV is approximately 62% of the ITV (r = 0.98) (A) and 91% of GTV (r = 0.95) (B). Data point in red denotes the patient’s treatment was re-planned due to significant reduction in tumor volume.

FCVs derived from CBCT were plotted with respect to the ITV and GTV generated by the treating physician from planning CT, revealing a strong linear correlation (r = 0.98 for ITV and r = 0.95 for GTV) (Fig 3). Since images from CBCT reflect those from average CTs (due to longer time of CBCT image acquisition), FCV calculated from CBCT is more strongly correlated to ITV. Numerically the FCV is approximately 62% of the ITV and 91% of GTV (Fig 3). The FCV is slightly smaller than the GTV because CBCT contouring was performed more conformally around the tumor than for treatment planning to more accurately capture smaller volume changes.

**Fig 4:**
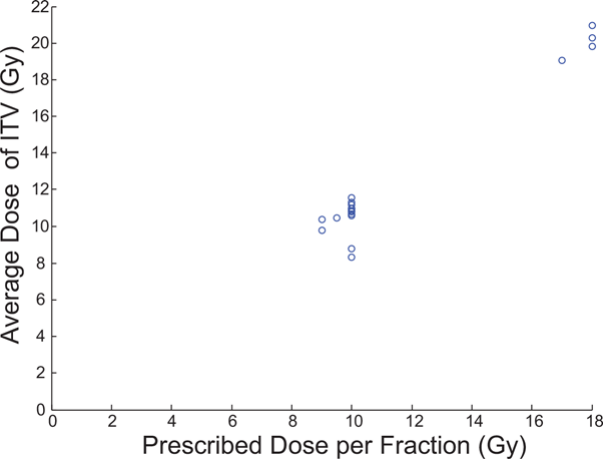
Physician prescribed dose vs average dose to ITV. The average ITV dose is calculated from the dDVH and is in agreement with the prescribed dose.

To ensure similar treatment plans between physicians at our institute, the average ITV dose was plotted with respect to the prescribed dose (Fig 4). The average ITV dose is in excellent agreement with the physician prescribed dose with the ITV volume receiving approximately 20% more radiation than the prescription dose (since ITV is contained within PTV and all treatment doses are prescribed to PTV).

### Tumor volume change during treatment

An average maximum volume increase of 15.1% (0% to 48.8%) and an average maximum volume decrease of 10.6% (0% to 70.8%) relative to the FCV was observed (S1 Fig). Significant tumor regression was noted in tumor III on CBCT, which was confirmed by repeat simulation CT, and adaptive treatment re-planning was performed for fraction 3–5 (see S1 Appendix for additional details). Because of this unusual change and the ensuing treatment re-planning, all statistical analyses are reported with the data from tumor III excluded; however, the inclusion of tumor III does not appreciably alter the strength of the reported correlations.

### Effect of tumor volume increase on dose coverage of tumors

**Fig 5:**
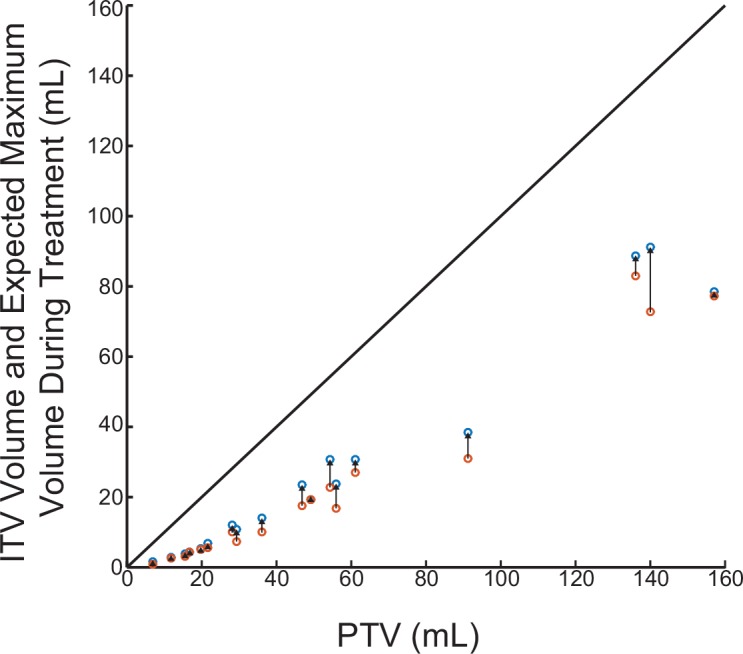
Expected volume increase on CT from original ITV volume with respect to PTV. Original ITV volume in orange and the expected volume increase on CT is in blue. The expected increase on CT was calculated using the 0.62 correlation factor from Fig 3. The diagonal line represents the maximum volume that can reside in the PTV.

Our analysis was not performed on the same imaging modality as the treatment planning and it is therefore difficult to precisely determine the effect on dose coverage; however, if the volume increase is assumed to occur uniformly and not alter the shape of the tumor, it is possible to approximate whether the tumor would extend beyond the PTV. Since the correlation between treatment planning ITV and FCV was significant, the maximum inter-fraction volume increase on CBCT can be converted to an expected inter-fraction volume increase on treatment planning CT using the 0.62 correlation factor calculated in Fig 3. The expected volume on treatment planning CT was plotted with respect to PTV (Fig 5). Using this method, the increase in volume of all tumors in our study is significantly less than the PTV volume and the volume increase is therefore expected to be contained within the PTV expansion (Fig 5).

### Correlation Analysis

#### FCV/ITV/GTV vs. CBCT volume change

**Fig 6:**
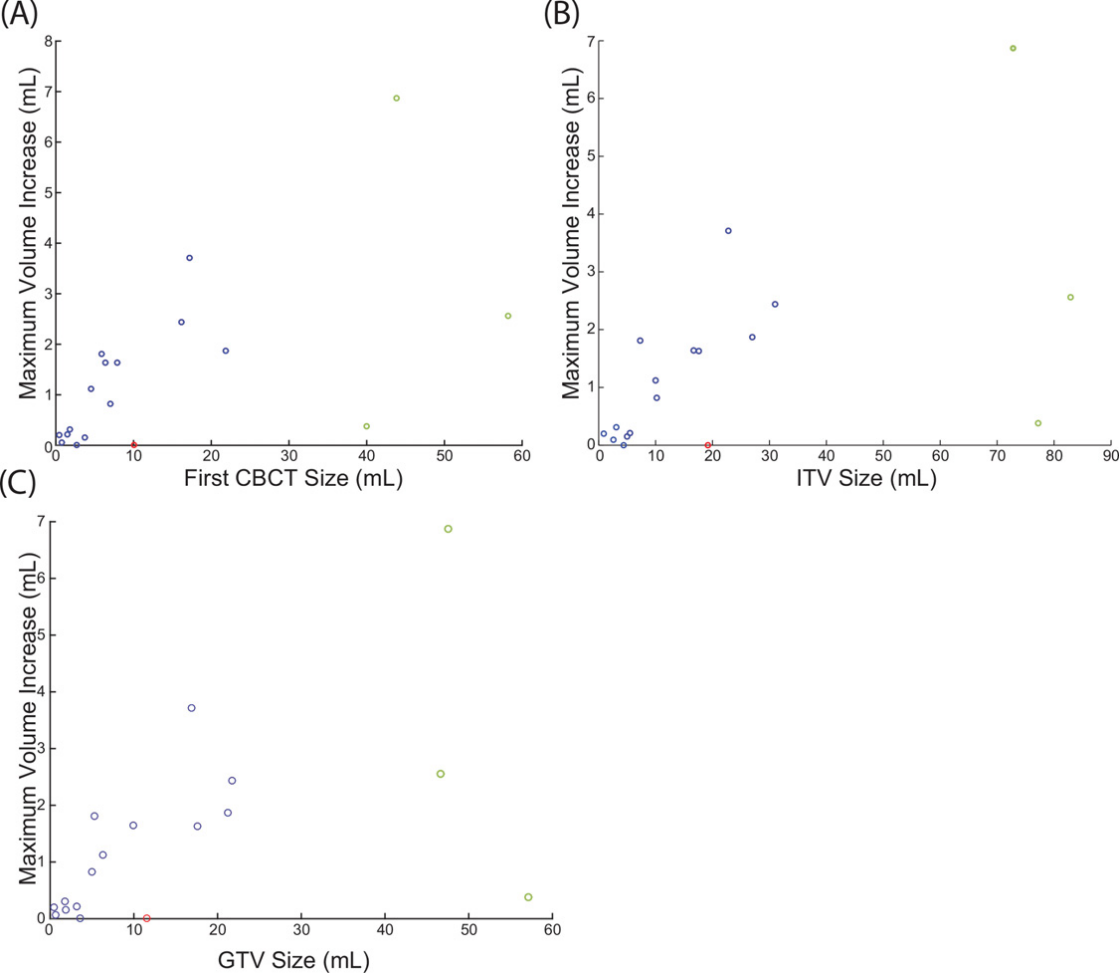
Linear regression between the FCV and maximum inter-fraction volume. Points in green were excluded from the linear regression due to their large volumes relative to the other points, but are included in the correlation analysis. Data point in red was excluded because the patient’s treatment was re-planned.

One of the most significant correlations is between the FCV (mL) and the maximum volume in CBCT (mL) during treatment (q = 0.0029); ITV and maximum volume (mL) (q = 0.0039); GTV and maximum volume (mL) (q = 0.0085). It is important to note that the correlation analysis is a non-parametric test, meaning the data has a trend but does not need to be linear to have a high correlation value. However, examining the data suggest there is a linear correlation for smaller tumors. Performing linear regression between the FCV and maximum volume during treatment (mL) revealed that the maximum tumor volume is approximately 13% bigger than the first CBCT contour (Fig 6A), 9.4% bigger than treatment planning ITV, and 11.6% bigger than treatment planning GTV volume. Three large tumors (greater than 22mL GTV) were excluded from the linear regression due to their large volumes; however, they are included in the correlation analysis above.

The relationship between minimum volume of tumors during treatment and FCV, ITV, GTV, or ITV ID per Fraction is nearly random (Table 3).

**Table 3:** Inter-fraction variables with significant correlation coefficients. Spearman correlation coefficient listed with q-values in parenthesis.

	***Maximum Volume (mL)***	***Minimum Volume (mL)***
**First cone-beam CT (FCV)**	0.8112 (q = 0.0029)	-0.1092 (q = 0.9846)
**ITV**	0.7966 (q = 0.0039)	-0.1219 (q = 0.9754)
**GTV**	0.7647 (q = 0.0085)	-0.0914 (q = 0.9846)
**ITV ID per Fraction**	0.7868(q = 0.0049)	-0.0660 (q = 0.9846)
	***Day of Maximum Volume***	***Fraction of Maximum Volume***
**Patient Age**	0.6133(q = 0.0972)	0.7274(q = 0.0122)

ITV, internal target volume; ID, integral dose

#### ITV ID per fraction vs. CBCT volume change

The ITV ID per fraction was directly correlated to the maximum volume (ml) (q = 0.0049). Neither the average dose delivered to the ITV nor the average dose of the non-PTV (presumably healthy lung tissue) was correlated to any inter-fraction tumor change parameter by a statistically significant margin (data not shown). These findings suggest the volume increase observed on CBCT requires the deposition of a large amount of energy at every fraction in the tumor.

#### Patient age vs. day/fraction maximum volume observed

Patient age at treatment onset was positively correlated to the fraction (p = 0.0009 and q = 0.0122) when the maximum tumor volume was observed (Table 3). The correlation between patient age and the day to maximum tumor volume did not reach statistical significance after multiple hypothesis correction but was statistically significant prior to multiple hypothesis (q = 0.0972 and p = 0.0088). To determine if these correlations are due to confounding variables, correlation analysis between patient age and all other parameters was performed, resulting in a statistically significant correlation only between non-PTV ID per fraction (healthy tissue irradiated) and patient age (p = 0.0458). Also, visual inspection suggested the presence of covariance between lung volume and patient age that did not reach a statistically significant threshold (p = 0.2010). To account for potential confounding covariance, a correlation analysis between patient age and day to maximum volume as well as fraction of maximum volume was repeated while controlling for both non-PTV ID per fraction and lung volume. Controlling for these variables resulted in a stronger correlation between age and the day of maximum volume (p = 0.0063), as well as a stronger correlation between age and treatment fraction of maximum volume (p = 0.0006). This suggests patient age is an independent predictor of the day when the maximum volume occurs, with respect to variables included in our study

#### Tumor histology vs. CBCT tumor volume parameters

A two-tailed t-test of the 7 volumetric parameter averages with respect to tumor histology was performed and did not reveal any statistically significant differences. Parameters having the strongest difference between squamous cell and adenocarcinoma histology are displayed in S1 Table.

## Discussion

Although several groups have previously demonstrated the presence of volumetric fluctuations during SBRT treatment of the lung, it is not clear if the observed inter-fraction changes are due to random fluctuations in volume, errors in tumor measurements, such as imaging artifacts, or represents a true biologic response, such as inflammation or tumor growth [12–18]

Progress in this area has been hampered by the absence of a simple and robust analysis method that accurately captures trends in both volume increase as well as decrease, especially when they occur during the same treatment course.

### A New Analysis Method

**Fig 7:**
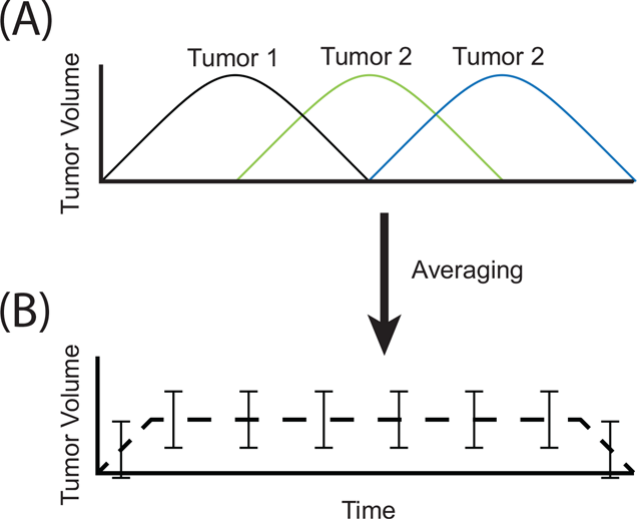
Loss of statistically significant correlations occurs when tumor volumes are averaged. If three tumors have the same volume increase during treatment yet occur at different times (A), simple averaging of the 3 volume time-courses will result in a curve with large standard error that significantly underestimates the volume increase (B).

In the present study we have employed a new methodology to quantify and classify volume changes during SBRT. One method that has been previously described is normalizing the volumetric changes by the initial volume and averaging the normalized volumes at each treatment fraction.[14, 16, 17] This method has two important limitations: only normalized volumes (%) can be analyzed and it assumes that volume changes occur at approximately the same time in different tumors (Fig 7). Using normalized volumes tends to bias the analysis toward smaller tumors since minor changes in volume correspond to large normalized volume changes. Secondly, the assumption that inter-fraction changes occur at similar timescales between different tumors is improbable and may therefore result in loss of statistically significant correlations (Fig 7). Our methodology ameliorates the above limitations. Simplifying the inter-fraction volume traces of each tumor to 7 parameters (Table 1 CBCT parameters) allows us to examine both normalized and non-normalized volumes. Furthermore, this allows us to perform correlation analysis of both the changes in tumor volume as well as the time when these changes occur, without assuming volume changes occur at the same time between different tumors

### Larger tumors increase in volume to a greater extent than smaller tumors

To our knowledge, this is the first report of inter-fraction volumetric changes during SBRT lung treatment being correlated to variables known at treatment onset. The presence of correlation between initial tumor volume and the maximum tumor volume as well as the lack of correlation between the initial tumor volume and minimum volume during treatment strongly suggests that the correlation between the initial tumor volume and maximum tumor volume is not an imaging artifact or random fluctuations in tumor volume. If the correlation was an imaging artifact or random fluctuations in tumor volume, we would expect either that the initial tumor volume would be correlated to both the maximum volume and the minimum volume since both a numeric increase in volume as well as decrease would be equally likely or we would expect no correlation at all.

The maximum inter-fraction tumor volume can be predicted from the FCV and an average maximum volume increase of approximately 13%, relative to the FCV, can be expected during SBRT treatment of the lung. Furthermore, the volume increase during treatment corresponds to an increase of 9.4% relative to ITV and 11.6% relative to GTV. However, it’s important to note that the volume increase could only be predicted for tumors with a GTV less than 22 mL in our study.

Our observed correlation may be due to characteristics of the tumor (ie size, histology, tumor biology) or radiation delivery parameters (ie dose, fractionation, ID). It is unclear whether the inability to predict the volume increase in larger tumors is due to biologic causes or simply because not enough large tumors were included in the study.

The maximum volume increase was not correlated to the treatment dose but it was correlated to the integral dose (ID) delivered to the ITV at every fraction. Since slightly different fractionation protocol was used in our study, we employed ID as one parameter to compare all tumors in our study. ID represents the energy deposited in the tumor and is a function of tumor volume, average dose, and some contribution from dose homogeneity. Since the ID generally scales with tumor volume, assuming uniform dose coverage, this finding does not distinguish whether the volume increase is independently linked to initial tumor volume or whether it is linked to increased radiation delivery. The similar correlation between maximum tumor volume and FCV as well as maximum tumor volume and ID delivered to the ITV, in addition to a lack of other correlations with these variables; suggest that initial tumor volume may independently predict volume increase during treatment, even in the presence of varying SBRT treatment protocols.

Using a different approach, Gunter *et al*. observed maximum inter-fraction volume of 115% and 110% in lung tumors treated with SBRT in 3 and 5 fractions respectively.[14] The cumulative increases and decrease of all tumors in the study were statistically significant; however, neither the maximum nor minimum volume changes could be predicted for individual tumors. It is important to note that the above study examined only the normalized data relative to FCV whereas the correlation between maximum volumes and FCVs was only apparent in our study when the volume changes were analyzed without normalization.

### Inter-fraction volume increase does not exceed the PTV

The observed volume increase is relatively small and tumors have significant room to expand before reaching PTV volume. Therefore the volume increase is unlikely to affect PTV coverage. One patient’s volume decreased significantly and his treatment plan was modified after the second fraction. Our experience showed that this is rare and tumor volume changes in most situations do not warrant adaptive re-planning.

### Patient age correlated to the fraction when maximum inter-fraction volume is observed

Patient age is correlated to the treatment fraction when the peak inter-fraction volume is observed. Our finding that repeating the correlation analysis between patient age while controlling for potential confounding variables (lung volume and non-PTV ID per fraction) results in stronger correlations was intriguing and requires further study. Specifically, it is not clear whether patient age is driving inter-fraction volumetric changes or if age indirectly affects treatment parameters that were not measured in this study.

Many biologic changes associated with the aging process may lead to this observation. For example, it has previously been hypothesized that inter-fraction volume increases during SBRT may be due to inflammation or edema.[14, 15] In the context of edema, the delayed onset of the volume increase in older age in our cohort may be explained by age-dependent dysregulation of the immune system.[20]

## Conclusion

Tumor volume fluctuations during SBRT of the lung were monitored by analyzing kV-CBCT images obtained before each treatment fraction, revealing both increases and decreases in tumor volume during treatment. Using our novel analysis method, we showed that these volume fluctuations were correlated to GTV, ITV, ITV ID per fraction, and patient age. In addition, volume increase for tumors less than 22mL could be predicted from treatment planning GTV or ITV. These findings suggest that inter-fraction volume fluctuations during SBRT treatment is not a random fluctuation or an imaging artifact but may reflects a biologic response and its clinical use should be further explored.

## Supporting Information

S1 Appendix Representative axial slices of tumor that increased in volume and additional discussion of the treatment course of tumor III. (DOCX)

S1 Fig KV-CBCT tumor volumes over time, day of each fraction, and radiation delivery parameters analyzed in the study. (DOCX)

S1 Table Two-tailed t-test of parameter averages with respect to tumor histology. Only parameters of general interest and those with low p-values are displayed.
(DOCX)
