# Supplementary material for: Inter-Fraction Tumor Volume Response during Lung Stereotactic Body Radiation Therapy Correlated to Patient Variables
Source: PLoS One. 2016 Apr 6;11(4):e0153245. doi: 10.1371/journal.pone.0153245 (PMC4822825; doi:10.1371/journal.pone.0153245)
Supplement: S1 Appendix — (DOCX) [file pone.0153245.s001.docx]

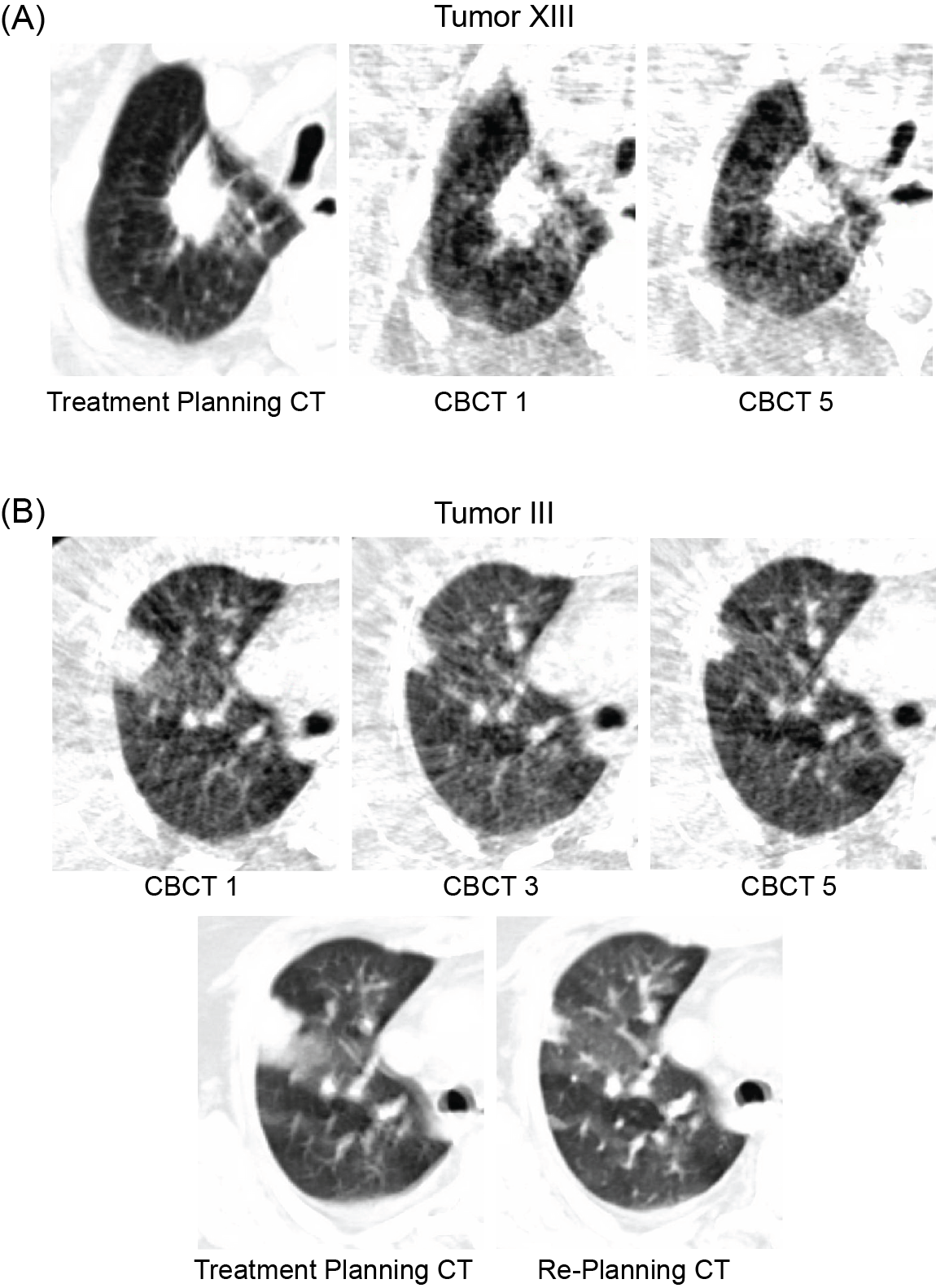


Representative images of a tumor that increased in volume during treatment is shown in (A). Tumor III had an unusually large reduction in tumor volume that required adaptive planning and a replanning CT was obtained at the 3^rd^ fraction (B). The original free-breathing GTV was 11.5 and ITV was 19.2 mL. A 4D CT was obtained during replaning revealed a free-breathing GTV of 6.7 mL and ITV of 10.2mL. The tumor continued to decrease until the final fraction. The patient has not been seen in clinic after completion of treatment and retrospective analysis of the medical record did not reveal any unique patient or treatment characteristics.
