# Supplementary material for: Inter-Fraction Tumor Volume Response during Lung Stereotactic Body Radiation Therapy Correlated to Patient Variables
Source: PLoS One. 2016 Apr 6;11(4):e0153245. doi: 10.1371/journal.pone.0153245 (PMC4822825; doi:10.1371/journal.pone.0153245)
Supplement: S1 Fig — (DOCX) [file pone.0153245.s002.docx]

|  |  | **CBCT Volume at each Fraction (mL)** | | | | | |  | |  | **Treatment Day at each Fraction** | | | | | |  |
| --- | --- | --- | --- | --- | --- | --- | --- | --- | --- | --- | --- | --- | --- | --- | --- | --- | --- |
|  |  | 1 | 2 | 3 | 4 | 5 |  | |  | | | 1 | 2 | 3 | 4 | 5 | |
| **Tumor** | I | 3.7 | 3.6 | 3.9 | 3.8 | 3.6 |  | | I | | | 0 | 1 | 4 | 8 | 11 | |
|  | II | 43.9 | 50.7 | 47.0 | - | - |  | | II | | | 0 | 3 | 7 | - | - | |
|  | III | 10.0 | 5.3 | 3.4 | 2.9 | 2.9 |  | | III | | | 0 | 3 | 6 | 8 | 10 | |
|  | IV | 17.2 | 20.9 | 19.8 | - | - |  | | IV | | | 0 | 2 | 7 | - | - | |
|  | V | 58.2 | 57.0 | 60.8 | 54.1 | 52.4 |  | | V | | | 0 | 2 | 4 | 7 | 9 | |
|  | VI | 4.5 | 5.0 | 5.6 | 4.5 | 3.7 |  | | VI | | | 0 | 2 | 4 | 7 | 10 | |
|  | VII | 0.8 | 0.5 | 0.8 | 0.6 | 0.8 |  | | VII | | | 0 | 2 | 4 | 7 | 10 | |
|  | VIII | 1.5 | 1.7 | 1.5 | 1.6 | 1.3 |  | | VIII | | | 0 | 2 | 5 | 7 | 9 | |
|  | IX | 40.0 | 39.8 | 38.1 | 38.7 | 40.4 |  | | IX | | | 0 | 2 | 6 | 8 | 10 | |
|  | X | 5.9 | 6.5 | 7.4 | 6.6 | 7.7 |  | | X | | | 0 | 2 | 6 | 8 | 10 | |
|  | XI | 0.4 | 0.5 | 0.5 | 0.5 | 0.6 |  | | XI | | | 0 | 2 | 6 | 8 | 10 | |
|  | XII | 1.8 | 1.9 | 1.8 | 1.8 | 2.1 |  | | XII | | | 0 | 2 | 6 | 8 | 10 | |
|  | XIII | 16.2 | 17.8 | 18.6 | 17.8 | 18.2 |  | | XIII | | | 0 | 2 | 4 | 7 | 9 | |
|  | XIV | 21.8 | 23.7 | 23.2 | 23.2 | 21.4 |  | | XIV | | | 0 | 3 | 6 | 8 | 13 | |
|  | XV | 7.0 | 7.8 | 7.7 | 6.9 | 6.5 |  | | XV | | | 0 | 2 | 7 | 9 | 15 | |
|  | XVI | 6.4 | 7.2 | 8.0 | - | - |  | | XVI | | | 0 | 9 | 14 | - | - | |
|  | XVII | 7.9 | 8.6 | 9.5 | 8.3 | 8.2 |  | | XVII | | | 0 | 4 | 6 | 8 | 11 | |
|  | XVIII | 2.7 | 2.0 | 2.3 | - | - |  | | XVIII | | | 0 | 7 | 10 | - | - | |

|  |  | **Radiation Delivery Parameters** | | | | |
| --- | --- | --- | --- | --- | --- | --- |
|  |  | ITV Average Dose per Fraction (cGy) | ITV ID per Fraction (cm^3^ x cGy) | nonPTV Average Cumulative Dose(cGy) | nonPTV Average Dose per Fraction (cGy) | nonPTV ID per Fraction (cm^3^ x cGy) |
| **Tumor** | I | 880 | 4399 | 50 | 10 | 14300 |
|  | II | 1980 | 144153 | 76 | 25 | 18233 |
|  | III | 1066 | 20459 | 773 | 155 | 204000 |
|  | IV | 1904 | 43415 | 879 | 293 | 533333 |
|  | V | 1084 | 89848 | 1101 | 220 | 396880 |
|  | VI | 1095 | 10954 | 668 | 134 | 178000 |
|  | VII | 1131 | 2941 | 597 | 119 | 216000 |
|  | VIII | 1038 | 5707 | 601 | 120 | 244000 |
|  | IX | 834 | 64392 | 869 | 174 | 158600 |
|  | X | 1118 | 8163 | 982 | 196 | 332000 |
|  | XI | 1057 | 951 | 982 | 196 | 332000 |
|  | XII | 1153 | 3575 | 516 | 103 | 171200 |
|  | XIII | 1078 | 33409 | 478 | 96 | 199000 |
|  | XIV | 1043 | 28159 | 708 | 142 | 150800 |
|  | XV | 1097 | 11193 | 710 | 142 | 234000 |
|  | XVI | 2028 | 33870 | 826 | 275 | 373333 |
|  | XVII | 972 | 17974 | 1250 | 250 | 206000 |
|  | XVIII | 2098 | 9230 | 684 | 228 | 366667 |
