# Supplementary material for: Inter-Fraction Tumor Volume Response during Lung Stereotactic Body Radiation Therapy Correlated to Patient Variables
Source: PLoS One. 2016 Apr 6;11(4):e0153245. doi: 10.1371/journal.pone.0153245 (PMC4822825; doi:10.1371/journal.pone.0153245)
Supplement: S1 Table — Only parameters of general interest and those with low p-values are displayed. (DOCX) [file pone.0153245.s003.docx]

| **Parameters** | **Squamous Cell Carcinoma** | **Adenocarcinoma** | **P-Value** |
| --- | --- | --- | --- |
| First Treatment CBCT Volume (mL) | 14.74 | 18.56 | 0.7104 |
| Minimum Inter-fraction Volume (mL) | -0.18 | -2.01 | 0.1417 |
| Maximum Inter-fraction Volume (mL) | 2.67 | 1.04 | 0.1054 |
| Minimum Inter-fraction Volume (%) | -8.80% | -7.86% | 0.8942 |
| Maximum Inter-fraction Volume (%) | 19.23% | 10.62% | 0.1196 |
|  |  |  |  |
| Day Minimum Volume Observed | 2.00 | 6.29 | 0.1125 |
| Treatment Fraction Minimum Volume Observed | 1.83 | 3.14 | 0.2057 |
| Normalized DVP of Treated Lung (cGy) | 868 | 1088 | 0.1917 |
| DVP of nonPTV Volume of Treated Lung (cm^3^ x cGy) | 613 | 863 | 0.1350 |
